# Vitamin D status in healthy black African adults at a tertiary hospital in Nairobi, Kenya: a cross sectional study

**DOI:** 10.1186/s12902-018-0296-5

**Published:** 2018-10-11

**Authors:** Elizabeth Kagotho, Geoffrey Omuse, Nancy Okinda, Peter Ojwang

**Affiliations:** 1 Department of Pathology, Aga Khan University Hospital Nairobi, P.O. Box 30270-00100, Nairobi, Kenya; 2 Department of Pathology, Maseno University, P.O. Box Private Bag, Maseno, Kenya

**Keywords:** Vitamin D deficiency, Cut-offs, Kenya, Africa

## Abstract

**Background:**

Vitamin D has been known since the twentieth Century for its benefits in bone health. Recent observational studies have demonstrated its benefits in infectious diseases such as tuberculosis and non-communicable diseases such as diabetes mellitus, cardiovascular diseases and cancer. This has led to a dramatic increase in testing among adults.

The cut-offs for vitamin D deficiency have been debated for decades and the current cut off is derived from a Caucasian population. Studies done among black African adults in Africa are few with vitamin D deficiency ranging from 5 to 91%. A few cut- offs have correlated vitamin D deficiency to physiological markers such as parathyroid hormone (PTH), calcium and phosphate with varying results.

**Methods:**

This was a cross sectional study carried out among blood donors at Aga Khan University hospital, Nairobi (AKUHN) from March to May 2015. Vitamin D (25(OH)D) levels were assayed and correlated with PTH, calcium and phosphate.

**Results:**

A total of 253 individuals were included in the final analysis. The proportion of study participants who had a 25(OH) D level of < 20 ng/ml thus classified as vitamin D deficient was 17.4% (95% C.I 12.73–22.07). The 25(OH) D level that coincided with a significant increase in PTH was 30 ng/ml.

Males were less likely to be vitamin D deficient (O.R 0.48 (C.I 0.233–0.993) p 0.04). Sunshine exposure for ≥3 h per day reduced the odds of being Vitamin D deficient though this was not statistically significant after multivariate regression analysis.

**Conclusions:**

We found a much lower prevalence of Vitamin D deficiency compared to many similar studies carried out in sub-Saharan Africa possibly due to the recruitment of healthy individuals and the proximity of Nairobi to the equator which allows for considerable exposure to sunshine. Vitamin D levels below 30 ng/mL was associated with a significant rise in PTH levels, suggesting that this cut off could be appropriate for defining Vitamin D deficiency in the population served by our laboratory.

## Background

Vitamin D is a prohormone, which is widely known for its role in bone health [1]. Observational studies in the last decade have implicated its role in reducing the risk of non-communicable diseases such as cancers, autoimmune diseases, cardiovascular diseases, disorders of glucose metabolism, neurodegenerative diseases and communicable diseases [2–4].

Due to the increasing evidence of the importance of vitamin D in relation to various health outcomes, many clinical laboratories in the USA have seen requests for vitamin D testing increase by up to 100% [5]. We have observed similar increments in the requests for vitamin D determination at the Aga Khan University Hospital, Nairobi laboratory.

Africa is a heterogeneous continent that straddles the equator and has northern and southern temperate zones. Most countries in the continent enjoy sunshine all year round due to their proximity to the equator [6]. Data on vitamin D levels among healthy Africans is scant with vitamin D deficiency ranging from 5 to 91% and mean vitamin D levels ranging from 4.4–46.1 ng/ml [7–10].

These studies however used different cut offs to define vitamin D deficiency making direct comparisons on its prevalence difficult. Majority did not correlate Vitamin D levels to surrogate markers of physiological deficiency such as calcium, phosphate and PTH and had insufficient sample sizes.

Other factors that influence the production of vitamin D3 by the skin include the melanin content of the skin, use of sunscreens, time of day, season, air pollution, cloud cover, age and the extent of clothing covering the body [11, 12]. Thus due to high melanin content (skin type V-VI) among Africans, vitamin D production is not as efficient as in light skinned individuals (skin type I-IV) [13]. However most African countries enjoy sunshine throughout the year. The use of sunscreens reduces the absorption of UVB radiation. Sun protection factor (SPF 30) reduces vitamin D3 synthesis by more than 95%. Increasing age reduces the amount of 7-dehydrocholesterol in the skin leading to less production of vitamin D3 [12]. Season, latitude and time of day affects the amount of solar UVB photons reaching the earth thus affecting vitamin D3 production by the skin [11].

We therefore sought to determine the vitamin D status of Kenyan blood donors of African origin and explore the possible risk factors for vitamin D deficiency. We also determined whether vitamin D deficiency as determined using published cut offs translated into a functional deficiency by assaying PTH, total calcium and inorganic phosphate.

## Methods

### Study design and site

This was a cross sectional study involving blood donors that was conducted at the AKUH,N Kenya blood donor unit from March to May 2015. AKUH,N is a private referral hospital that caters for the residents of Nairobi, Kenya and the greater East African region and receives 400–500 blood donors per month. Nairobi is located at latitude 1^0^18’S and experiences sunshine throughout the year due to its proximity to the equator.

### Study participants

Blood donors of African origin between the ages of 18–65 years who met the criteria of blood donation as per the hospital policies were eligible to join the study. It was assumed that those who met the strict criteria for qualifying as a blood donor were healthy. Consecutive sampling was used until the desired sample size was achieved. Blood donors with a positive HIV, hepatitis B, hepatitis C, malaria or syphilis result were excluded from the final analysis.

### Data collection

Upon obtaining an informed written consent, the study participants filled a questionnaire. Its purpose was to collect information regarding sun exposure, which was expressed in terms of the number of hours spent outside, dietary intake of vitamin D rich foods such as oily fish and dietary intake of calcium and phosphate rich foods. This was in addition to the blood donor screening questionnaire that checks whether one is fit to qualify as a blood donor based on appropriate age, weight, haemoglobin level, exposure to infectious diseases and medication intake.

### Sample collection and processing

Blood samples were collected in EDTA tubes for PTH and 25(OH) D and serum clot activator tubes for total calcium, inorganic phosphate and albumin. Centrifugation was done immediately at 4000 rpm for 5 min. Plasma and serum was then aliquoted into 2 ml plastic tubes and stored at -20 °C and analysed in batches of 50.

### Sample analysis

All samples were analysed on the ROCHE COBAS ® platform e601 for PTH, 25(OH) D and e501 for total calcium, inorganic phosphate and albumin. Analysis of 25 (OH) D was done using an electrochemiluminescent competitive immunoassay assay and reported in nanograms per milliliter (ng/ml). PTH was analysed using an electrochemiluminescent sandwich immunoassay and reported in picograms/milliliter (pg/ml). Serum calcium and phosphate levels were measured photometrically using the o-cresolphthalein complexone and molybdate methods respectively and reported in mmol/l. Serum albumin was analysed using a calorimetric assay and reported in in g/l.

Daily quality controls were within acceptable ranges. All analytes had been registered under the Randox International Quality Assessment Scheme (RIQAS) with acceptable running means standard deviation index of − 0.21,-0.80,-0.44,-0.30,0.03 for 25 (OH) D, PTH, Calcium, phosphate and albumin respectively.

Vitamin D levels were categorized as sufficient (> 30 ng/ml), insufficient (21-29 ng/ml) and deficient (< 20 ng/ml) based on the IOM classification that has been widely adopted globally. The reference ranges for PTH were 15-65 pg/ml, total calcium 2.1–2.66 mmol/l, inorganic phosphate 0.84–1.45 mmol/l. Corrected calcium was calculated for all study participants who had a low albumin level and this was used in the final analysis.

### Statistical analysis

Data was analysed using IBM® SPSS® Statistics V22.0. The prevalence of vitamin D deficiency was expressed as a percentage with 95% confidence intervals (CI). Continuous variables i.e. 25(OH)D, PTH, phosphate and calcium were presented in form of medians and inter quartile ranges.

A scatter plot was used to determine the relationship between vitamin D and PTH and a quadratic equation was used to arrive at the level of vitamin D beyond which a decline resulted in an increase in PTH levels. This was determined using the point of inflection on the graph.

Man Whitney U test was used to compare medians or mean ranks of calcium and phosphate levels between the vitamin D deficient (25(OH)D levels of < 20 ng/ml) and non-deficient group (25(OH)D levels of ≥20 ng/ml). Binary logistic regression and multivariate regression analysis was used to calculate crude and adjusted odds ratios for risk factors of vitamin D deficiency. These risk factors included sunshine exposure in hours, gender and dietary intake of oily fish. Age was not adjusted for, since there was no statistically significant difference between the vitamin D deficient and non-deficient group. A *p* value < 0.05 was considered statistically significant.

## Results

A total of 258 participants were recruited into the study of whom 5 were excluded from the final analysis due to a positive screen for HIV, Hepatitis B or C virus.

The median (IQR) age of the study participants was 33.07(40) years. Overall 62 (24.5%) of the study participants were female.

Table 1 shows the baseline demographic and biochemical variables of the study participants.

**Table 1:** Descriptive characteristics of the study participants

	Male (*n* = 191)		Female (*n* = 62)		Total (*n* = 253)		Male vs female
	Median(IQR)	Min-Max	Median (IQR)	Min-Max	Median (IQR)	Min-Max	*p*- value
Age (years)	33 (40)	19–59	31 (30)	21–51	33.07 (40)	19–59	0.304
Vitamin D (ng/ml)	28.24 (44.47)	8.89–53.36	25.08 (34.12)	8.57–42.71	27.80 (44.79)	8.57–53.36	0.002
PTH (pg/ml)	36.46 (72.69)	10.38–83.07	36.71 (83.38)	16.72–100.1	36.7 (89.72)	10.38–100.1	0.534
Calcium (mmol/l)	2.42 (1.50)	1.23–2.73	2.38 (0.72)	2.05–2.77	2.41 (1.54)	1.23–2.77	0.168
Phosphate (mmol/l)	1.17 (3.32)	0.66–3.98	1.17 (2.58)	0.82–3.40	1.13 (3.32)	0.66–3.98	0.360

*IQR* interquartile range

### Vitamin D status

**Fig. 1:**
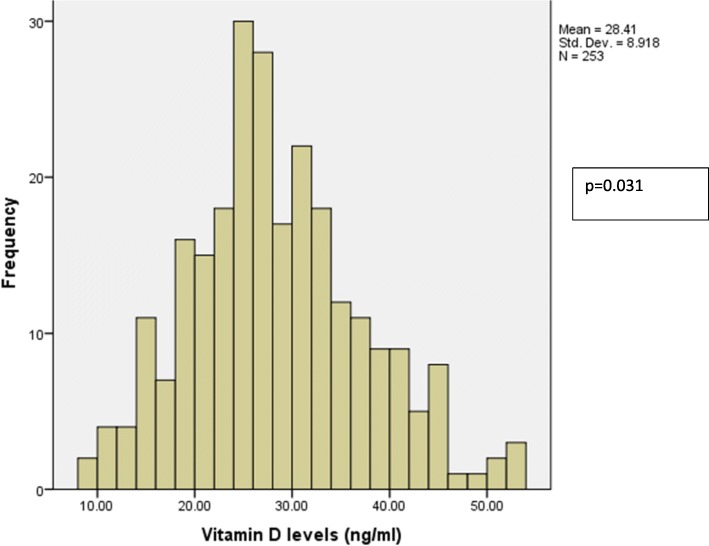
Distribution of vitamin D levels

Study participants who were classified as vitamin D deficient (levels < 20 ng/ml) was 17.4% (95% C.I 12.73–22.07). Those classified as vitamin D insufficient were 42.6% (95% C.I 36.51–48.69) while 40% (95% C.I 33.96–46.04) were classified as vitamin D sufficient. Figure 1 shows the distribution of vitamin D levels.

Males were less likely to be vitamin D deficient (O.R 0.481(C.I 0.233–0.933) *p* = 0.04). Sunshine exposure for > 3 h significantly reduced the odds of one been vitamin D deficient however this was not statistically significant after multivariate regression analysis. The use of sunscreens and dietary intake of oily fish did not significantly increase or reduce the odds of one been vitamin D deficient respectively though the numbers in these categories were very small as shown in Table 2.

**Table 2:** Comparison of risk factors for Vitamin D deficiency

	Deficient	Non-deficient	*P* value	Crude O.R (C.I)	*P* value	Adjusted O.R. (C.I)
Gender: N (%)						
Female^a^	17 (27.4%)	45 (72.6%)				
Male	27 (14.1%)	164 (85.9%)	0.03	0.463 (0.212–0.784)	0.04	0.481 (0.233–0.933)
Sunshine exposure: N (%)						
≤ 1 h daily^a^	66 (76.7%)	20 (23.3%)				
1–3 h daily	57 (81.4%)	13 (18.6%)	0.48	0.753 (0.344–0.1647)	0.13	0.893 (0.0.658–4.912)
> 3 h daily	86 (88.7%)	11 (11.3%)	0.03	0.422 (0.189–0.942)	0.08	0.623 (0.487–3.889)
Oily fish intake: N (%)						
None^a^	30 (19.9%)	121 (80.1%)				
≥ once a week	14 (13.7%)	88 (86.3%)	0.20	0.642 (0.231–1.281)		
Sunscreen use: N (%)						
None^a^	42 (17.6%)	197 (82.4%)				
Yes	2 (14.3%)	12 (85.7%)	0.75	0.782 (0.169–3.623)		

^a^Reference group, *O.R* Odds ratio, *C.I* Confidence interval

**Fig. 2:**
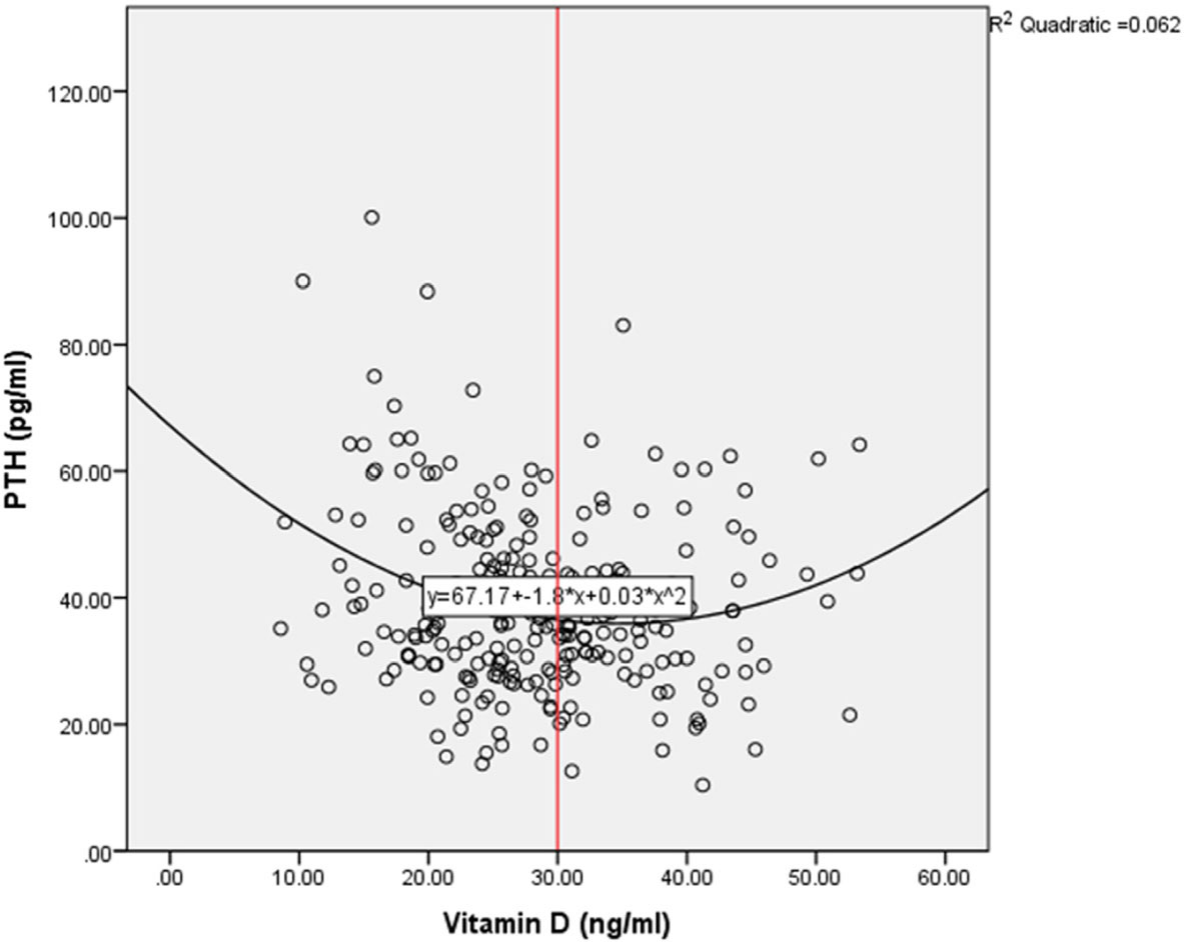
Scatter plot showing the relationship of vitamin D and PTH

There was an inverse relationship between vitamin D levels and PTH and the vitamin D level that coincided with a significant increase in PTH was 30 ng/ml as shown in Fig. 2.

There was no statistically significant difference in calcium and phosphate levels between the vitamin D deficient and non- deficient individuals.

## Discussion

The prevalence of vitamin D deficiency was 17.4% (95% C.I 12.73–22.07). This study population was representative of a healthy black African population in Kenya and despite being vitamin D deficient they did not have any related symptomatology. Furthermore, there was no statistically significant difference in calcium and phosphate levels between the vitamin D deficient non-deficient individuals. This puts into question whether levels below 20 ng/mL should trigger a medical intervention in the absence of related symptomatology.

The prevalence we found in our study is in stark contrast to a study done by Luxwolda et al. in Tanzania on the Hadzabe’s and Maasai’s who had a mean vitamin D of 46.1 ng/ml with none of the participants being vitamin D Deficient [8]. The difference in vitamin D levels might have been due to the fact that Maasai’s and Hadzabe’s are pastoralists’, hunters and gatherers and spend most of their time outdoors with minimal clothing.

The US National Nutrition and Health survey (NHANES) III reported that at least 53–76% of African Americans were vitamin D deficient during winter [14] and Powe et al. reported a mean vitamin D level of 15 ± 0.2 ng/ml among 1181 black Americans [15]. The reasons cited for this level of deficiency included dark skin colour thus resulting in inefficient vitamin D production by the skin and lack of regular vitamin D supplementation.

We found that females were more likely to be vitamin D deficient. Possible explanations for this observation include increased subcutaneous adiposity and higher Body mass index (BMI) among females resulting in increased 25(OH) D storage in adipose tissue thus not readily available for conversion to calcitriol [16]. Subcutaneous adiposity and BMI were however not taken for the study participants.

Studies have been done correlating vitamin D to physiological markers of vitamin D deficiency such as PTH, calcium and phosphate. These studies have reported that PTH is inversely associated with vitamin D and begins to plateau at a vitamin D level between 16 and 40 ng/ml [17–19]. This is the lowest vitamin D level beyond which PTH starts rising. Whether these values would be suitable cut-offs still remains controversial. The US Institute of Medicine 2011 guidelines quoted that one of the reasons behind the current recommendation of the cut off 20 ng/ml to define deficiency was the lowest vitamin D level at which PTH started rising [17]. From our study, the derived vitamin D cut-off based on the point of inflection i.e. the vitamin D level when PTH starts rising is 30 ng/ml. This was similar to a cross-sectional study done by Nusrat et al. in Kenya, unpublished data who looked at vitamin D levels and PTH in exclusively 97 breast fed infants and came up with a cut-off of 30 ng/ml. If a cut off of 30 ng/ml was adopted then 60.1% (95% C.I. 54.07–66.13%) of the study population would have been vitamin D deficient.

This raises the question whether there are other factors such as bioavailable vitamin D and vitamin D binding protein that should be considered when interpreting 25(OH) D levels since this was essentially a healthy population. This was reported in a study by Powe et al. where Black Americans compared to whites had low Vitamin D levels (15.6 ± 0.2 ng/ml vs. 25.8 ± 0.4 ng/ml, *P* < 0.001) and vitamin D binding protein (168 ± 3 μg/ml vs. 337 ± 5 μg/ml, *P* < 0.001) but similar concentrations of bioavailable Vitamin D 2.9 ± 0.1 ng/ml vs 3.1 ± 0.1 ng/ml, *P* = 0.71. Genetic polymorphisms partially explained the differences in Vitamin D binding protein in the two populations [15].

Sunshine exposure for ≥3 h was associated with a decreased likelihood of being vitamin D deficient as compared to < 1 h. This was however not statistically significant using multiple logistic regression. More than 95% of vitamin D 3 is from the sun [20]. Nairobi lies at latitude 1018’S thus enjoying sunshine all year round. Increased skin pigmentation reduces the production of vitamin D 3 on the skin. Since most Africans are skin type V and VI longer sunshine exposure would be required to make vitamin D 3.

Sunscreen use increases the risk of vitamin D deficiency, with sunscreens with an SPF of 30 and above reducing vitamin D production by upto 95% [20]. From this study 14 out of the 253 study participants reported the use of sunscreen or sunscreen containing lotions and there was no statistically significant difference in vitamin D levels between those who did and didn’t use sunscreen. This study was however not sufficiently powered to test for this association.

### Limitations

This study was not sufficiently powered to show significant differences for the different risk factors and as such, most of the analysis was exploratory. The absence of statistically significant differences could be due to the small sample size, which increases the risk of a type II error.

Sun exposure, use of sunscreens and dietary intake of vitamin D and calcium rich foods data collected for this study was self-reported thus liable to a reporting bias.

Skin type for the study participants was also not determined. Potentially, variation in skin types could influence the impact of some risk factors on Vitamin D levels.

Factors that can affect vitamin D levels such as Vitamin D supplementation, Body Mass Index (BMI) and subcutaneous adiposity were not taken.

## Conclusion

This study highlights that one in every six healthy Kenyan adults of African origin donating blood at AKUHN is vitamin D deficient using the cut-off of 20 ng/ml. These adults had no signs and symptoms of deficiency and biochemically appeared not to have any significant abnormality. Clinicians need to be aware of the level of deficiency in the healthy population so that they can take this into account when interpreting Vitamin D results in patients. Whether such individuals should have vitamin D supplementation to boost their levels above 30 ng/mL is a question that still needs to be answered. Vitamin D testing should also be restricted to patients at risk as per the Endocrine society 2011 guidelines. This includes patients with osteoporosis, chronic kidney disease, pregnant and lactating mothers, hyperparathyroidism and malabsorption syndromes.

Studies being carried out linking vitamin D to various health outcomes also need to bear in mind the baseline level of deficiency in the healthy Kenyan population.

Suitable 25 (OH) D cut-offs need to be established in the African population and these need to be predictive of specific well defined outcomes. Since total vitamin D and not the active form is what is routinely assayed, studies correlating 25(OH)D levels, vitamin D binding protein and physiological markers of vitamin D deficiency among Africans need to be done.

## Abbreviations

25(OH)D: 25 hydroxyvitamin D
AKUHN: Aga Khan University Hospital Nairobi
BMI: Body Mass Index
EDTA: Ethylenediaminetetraacetic acid
IOM: Institute of medicine
NHANES: National Health and Nutrition Examination Survey
PTH: Parathyroid hormone
RIQAS: Randox International Quality Assessment Scheme
SPF: Sun protection factor
UVB: Ultraviolet Radiation B
VDBP: Vitamin D binding protein
